# Contribution of individual excitatory synapses on dendritic spines to electrical signaling

**DOI:** 10.3389/fnins.2025.1620654

**Published:** 2025-07-29

**Authors:** Ju-Yun Weng, Cesar Ceballos, Dejan Zecevic

**Affiliations:** Department of Cellular and Molecular Physiology, Yale University School of Medicine, New Haven, CT, United States

**Keywords:** spines, dendrites, synapses, AMPA, voltage imaging

## Abstract

Dendritic spines, ∼1 μm protrusions from neuronal dendrites that receive most of the excitatory synaptic inputs in the mammalian brain, are widely considered the elementary computational units in the nervous system. The electrical signaling in spines is not fully understood, primarily for methodological reasons. We combined the techniques of whole-cell recording and voltage imaging to study excitatory postsynaptic potentials evoked by two-photon glutamate uncaging (uEPSPs) on individual dendritic spines on basal dendrites in rat cortical slices. We analyzed the initiation, temporal summation, and propagation of uEPSPs from the spine head to the parent dendrites in three principal neocortical pyramidal neuron classes. The data show no significant attenuation of uEPSPs across the spine neck in most tested mushroom spines on basal dendrites. This result implies that synapses on examined spines are not electrically isolated from parent dendrites and that spines do not serve a meaningful electrical role. Using the same imaging techniques, we characterized the temporal summation of uEPSPs induced by repetitive glutamate uncaging, mimicking the burst activity of presynaptic neurons. We found that summing responses to high-frequency repetitive quantal EPSPs is strictly limited in amplitude and waveform. This finding reveals a biophysical mechanism for preventing synaptic saturation.

## Introduction

This study aimed to define the electrical role of dendritic spines and characterize the contribution of single spine synapses to the electrical signaling of individual neurons. Several studies hypothesized that dendritic spines serve a unique electrical role because spine head synapses are electrically isolated from the parent dendrite by the slender spine neck. Other studies concluded that spine synapses are not electrically isolated and that spines have no electrical role. At present, neither one of these opposing postulates is universally accepted. The hypothetical electrical role of spines is a critical issue because it implies high electrical resistance of the ∼1 μm long spine neck cable (R_*neck*_) relative to the input impedance (Z_*dendrite*_) of the ∼ 60–1,000 μm long parent dendritic cables. The postulated high value of R_*neck*_ would, in turn, imply the functional significance of the highly variable morphology of individual spine necks, making practically every spine functionally different.

One group of studies supporting the electrical role of spines postulates high R_*neck*_ partially or entirely on theoretical grounds using numerical simulations (Koch and Poggio, 1983; Bloodgood and Sabatini, 2005; Grunditz et al., 2008; Bloodgood et al., 2009; Gulledge et al., 2012; Xu et al., 2012; Araya et al., 2014; Tønnesen et al., 2014; Acker et al., 2016; Cartailler et al., 2018; Lagache et al., 2019). Another group of studies postulates high R_*neck*_ based on indirect measurements of Ca^2+^-signals from which they derived membrane potential transients in dendritic spines that could not be recorded directly. A different indirect approach results interpreted to support high R_*neck*_ measured diffusional resistance of the spine neck experimentally and derive electrical resistance theoretically (Bloodgood and Sabatini, 2005; Araya et al., 2006b,2006a,^2014^; Grunditz et al., 2008; Bloodgood et al., 2009; Harnett et al., 2012; Tønnesen et al., 2014; Bywalez et al., 2015; Acker et al., 2016; Hage et al., 2016; Beaulieu-Laroche and Harnett, 2018). Finally, a third group of studies postulated high R_*neck*_ based on an attempt to directly probe spine membrane potential changes using electrical and optical techniques (Jayant et al., 2016; Kwon et al., 2017; Cartailler et al., 2018; Cornejo et al., 2022).

In contrast, a different set of reports provides indirect evidence that, in most spines (> 80%), the neck resistance is too small relative to the input impedance of the dendrite to affect synaptic signals. Some of these studies are based on theoretical considerations and numerical simulations (Rall and Rinzel, 1973; Rall, 1974; Koch and Zador, 1993). Other studies in this group are based on experimental measurements of diffusional resistance of the spine neck, which indicated relatively low spine neck resistance in the majority of spines (Svoboda et al., 1996; Takasaki and Sabatini, 2014; Tønnesen et al., 2014; Miyazaki and Ross, 2017, 2022). Previously, we provided direct evidence for the low electrical resistance of the spine neck as recorded from individual spines on basal dendrites in one class of principal pyramidal neurons. The data were acquired using voltage-sensitive dye recordings with adequate sensitivity and spatiotemporal resolution (Popovic et al., 2015a). This technique uses an organic voltage-sensitive dye that acts as a transmembrane voltmeter with a linear scale in the physiological range of neuronal membrane potential signals. The traces showing fluorescent light intensity changes from the voltage-sensitive probe precisely track the membrane potential transients. The spatial resolution of this method allowed monitoring of uEPSP voltage transients simultaneously at both ends of the spine neck, i.e., in the spine head and the parent dendrite at the base of the spine. In this study, we improved the temporal resolution of voltage imaging, confirmed earlier conclusions by additional measurements from L5 pyramidal neurons, and extended the experiments to the spines of two other classes of principal pyramidal cells (L2/3 and L6). Under the described recording conditions, the interpretation of the results did not require any specific assumptions. The obtained data argue for a minimal or no impact of spine necks on electrical signaling in most cases of sampled mushroom spines on basal dendrites of all three classes of neurons. Using the same technique, we monitored the local effects of repetitive activation of individual excitatory synapses and revealed the biophysical mechanism that minimizes synaptic saturation.

## Materials and methods

### Experimental design and statistical analysis

We used high-sensitivity voltage imaging with an organic electrochromic dye to analyze the electrical role of dendritic spines in selectively labeled L2/3, L5, and L6 pyramidal neurons in rat cortical slices. Statistical analyses were performed using GraphPad Prism 9.3.1. All values are reported as mean ± SEM.

### Slices, patch-clamp recording, and intracellular application of dyes

All surgical and experimental procedures followed the Public Health Service Policy on Humane Care and Use of Laboratory Animals and were approved by the Yale University Institutional Animal Care and Use Committee. Experiments were carried out on somatosensory cortex slices from 18 to 30 days old rats of either sex. The animals were decapitated following deep isoflurane anesthesia, the brain was quickly removed, and 300 μm thick coronal cortical slices were cut in an ice-cold solution using a custom-made rotary slicer with a circular blade (Specialty Blades Inc., Staunton, VA). Slices were incubated at 34°C for ∼30 min and then maintained at room temperature (23–25°C). The standard extracellular solution used during recording contained (in mM): 125 NaCl, 25 NaHCO_3_, 20 glucose, 2.5 KCl, 1.25 NaH_2_PO_4_, 2 CaCl_2_, and 1 MgCl_2_, pH 7.4 when bubbled with a 5% CO_2_ gas mixture balanced with 95% O_2_. Somatic whole-cell recordings in the current clamp or voltage-clamp mode were made with 4–6 MΩ patch pipettes using a Multiclamp 700B amplifier (Axon Instruments Inc., Union City, CA). Voltage-clamp recordings were made with series resistance compensation set at 70%. The pipette solution contained (in mM): 120 K-gluconate, 3 KCl, 7 NaCl, 4 Mg-ATP, 0.3 Na-GTP, 20 HEPES, and 14 Tris-phosphocreatine (pH 7.3, adjusted with KOH) and 0.8 mM of the voltage-sensitive dye JPW3028 (Antić and Zecević, 1995). This electrochromic voltage-sensitive dye tracks the membrane potential exactly with the response time constant of less than 2 ms (Salzberg et al., 1993). The pharmacological agents were obtained from Tocris. The somatic whole cell recording data were not corrected for liquid junction potential. We selected pyramidal cells with intact dendrites in one plane of focus close to the surface of the slice (to minimize light scattering) using infrared differential-interference contrast (DIC) video-microscopy. The recordings were from mushroom spines on superficial basal dendrites at different distances from the soma (60–230 μm). Stubby spines without clearly defined spine necks were excluded. This study was restricted to basal dendrites because back-propagating action potentials (bAPs) used for normalizing the sensitivity of optical recordings from different locations do not propagate into all parts of the apical dendritic arbor. Individual pyramidal neurons were labeled with the membrane impermeant voltage-sensitive dye by allowing free diffusion of the probe from the somatic patch pipette in the whole-cell configuration. We used a voltage probe for an intracellular application, JPW3028, which was synthesized and provided by Leslie Loew, Centre for Cell Analysis and Modeling, UConn Health Centre. This dye is a close analog of JPW1114 (Zecević, 1996) with similar voltage sensitivity available from Invitrogen as D6923. Glass pipettes were first filled from the tip with the dye-free solution by applying negative pressure for about 15 s and then back-filled with the solution containing the indicator dye (0.8 mM). Intracellular staining was accomplished by free diffusion of the dye from patch electrodes in 15–60 min, depending on electrode access resistance. After enough dye diffused into the cell body, as determined by measuring resting fluorescence intensity from the soma, the patch electrode was detached from the neuron by forming an outside-out patch. The staining level was determined empirically as a compromise that attains an adequate level of fluorescence without causing damage by prolonged dialysis from the patch pipette. The preparation was typically incubated for an additional 1.5–2 h at room temperature to allow the voltage-sensitive dye to spread and equilibrate in the dendritic arbor. To obtain electrical recordings, the cell body was re-patched using an electrode filled with the dye-free intracellular solution before making optical measurements at 34°C. Both APs and steady-state hyperpolarizing signals were evoked by transmembrane current pulses delivered via the recording electrode attached to the soma in whole-cell configuration (Popovic et al., 2012).

### Optical recording

The recording setup was built around a stationary upright microscope (Olympus BX51; Olympus Inc., United States) equipped with a high spatial resolution CCD camera for infrared DIC video microscopy (CCD-300-RC, Dage-MTI, Michigan City, IN, United States) and a high-speed data acquisition camera used for voltage imaging. This camera (NeuroCCD-SM, RedShirtImaging LLC, Decatur, GA, United States) is characterized by relatively low spatial resolution (80 × 80 pixels), exceptionally low read noise, and a full frame rate of 2 kHz. The frame rate can be increased to 5 kHz by reading out the central subsection of the camera chip of 24 × 80 pixels. The 5 kHz recording mode was used in most experiments to reconstruct signals accurately for calibration and comparison. The brain slice was placed on the microscope stage. A water-dipping objective projected the stained neuron’s fluorescent image onto the CCD positioned in the primary image plane of the microscope. We used a 100X/1.0 NA Olympus objective for optical recordings from individual spines. This objective was a compromise between imaging area, spatial resolution, and signal-to-noise ratio (S/N). The optical recording was carried out in the wide-field epifluorescence microscopy mode. A frequency-doubled 500 mW diode-pumped Nd: YVO4 continuous wave (CW) laser emitting at 532 nm (MLL532, Changchun New Industries Optoelectronics Tech. Co., Ltd., Changchun, China) was the source of excitation light. The laser beam was directed to a light guide coupled to the microscope via a single-port epifluorescence condenser (TILL Photonics GmbH, Gräfelfing, Germany) designed to provide approximately uniform illumination of the object plane. The laser was used as a light source in place of a conventional Xenon arc-lamp to increase the sensitivity of Vm-imaging by (1) providing a monochromatic excitation light at the red edge of the absorption spectrum to maximize the Vm sensitivity of the dye (Loew, 1982; Kuhn et al., 2004; Holthoff et al., 2010; Popovic et al., 2012, 2015a) and (2) increasing the intensity of the excitation light beyond the level that an arc-lamp can achieve. Excitation light was reflected to the preparation by a dichroic mirror with a central wavelength of 560 nm. The fluorescence light was passed through a band pass emission filter (FF01-720/SP-25; 720 nm blocking edge BrightLine multiphoton short-pass emission filter, Semrock). The laser light was gated for voltage imaging by a high-speed shutter (Uniblitz LS6, driver D880C). Data acquisition and analysis were carried out using NeuroPlex software (RedShirtImaging). In this configuration, a CCD frame (80 × 80 pixels) corresponded to a field of 18 × 18 μm in the object plane, with each pixel receiving light from an area of 0.23 × 0.23 μm in the focal plane.

### Computer-generated holography for two-photon uncaging of glutamate

The voltage imaging setup was integrated with an ultra-fast pulsed titanium-sapphire laser tuned to 720 nm for two-photon glutamate uncaging (Chameleon Ultra, Coherent Inc.). The light intensity of the laser and the duration of the uncaging pulse were controlled by a Pockells cell (Model 350-80, Conoptics Inc.). The two-photon uncaging spot was generated using a commercial module for holographic illumination (Phasor–3i Intelligent Imaging Innovation, Inc., Denver, CO, United States), modulating a 720 nm laser source controlled by SlideBook software (3i Intelligent Imaging Innovation). We used multipoint patterns acquired on the NeuroCCD camera to calibrate the exact positioning of the holographic spots in the field of view. It was possible to achieve submicron precision in spot positioning by introducing a correcting stretch, translation, and rotation transformation to the input patterns provided by the Phasor algorithm. The size of the two-photon 720 nm uncaging spot was measured at the focal plane of the microscope objective illuminated by the beam of parallel light overfilling its back opening. The light was focused on the thin film of rhodamine 6G spin-coated on a coverslip. The induced fluorescence spot was projected onto a CCD camera chip positioned in the primary image plane of the microscope, and its size was measured to be ∼0.6 μm in diameter (Supplementary Figure S1). The holographic illumination permitted a remote sub-micrometer repositioning of the uncaging spot mediated by the liquid crystal Spatial Light Modulator (SLM). This feature greatly facilitates necessary corrections due to preparation movements during temporal averaging. The exact focal volume of two-photon excitation (Matsuzaki et al., 2001) was not determined, but the spatial resolution of glutamate uncaging was sufficient to activate one spine synapse in isolation. This spatial resolution has been repeatedly documented (Matsuzaki et al., 2001; Smith et al., 2003; Carter and Sabatini, 2004; Sobczyk et al., 2005; Tazerart et al., 2020). However, we did find that, in several cases, the 720 nm short uncaging pulse of red light scattered from the focal point reduced the intensity of the voltage-sensitive dye fluorescence transiently for a fraction of a millisecond. Following the uncaging pulse, the intensity instantaneously (on a biological time scale) returned to the base level. The photobleaching of the dye could be safely excluded as a cause of this reduction because the uncaging 720 nm red light is outside the absorption spectrum of the fluorescent styryl dye JPW302. Additionally, the bleaching effect is irreversible. Thus, the remaining possibility is that the reduction was caused by the Stimulated Emission Depletion (STED). Because we positioned the uncaging light spot at about 0.5 μm away from the spine, because the duration of the uncaging pulse was very short (0.2–0.4 ms), and because the STED effect is instantaneous on the biological time scale, only one data point was effected (Supplementary Figure S2). Thus, the STED effect did not alter the shape and peak amplitude of uEPSPs. Wide field illumination was used to obtain an image of dye-loaded dendrites and identify structures of interest for glutamate uncaging and voltage imaging. DNI-glutamate TFA provided by Femtonics KFT (Budapest, Hungary), which has ∼7 times higher two-photon uncaging efficiency (Chiovini et al., 2014) than the commonly used MNI caged compound, was bath applied at a concentration of 4 mM. The illumination spots were placed at a distance of ∼ 0.5 μm from individual spine heads. The precise spatial relationship between the uncaging spots and spine heads was uncertain at the sub-micrometer spatial scale because of light scattering in the brain tissue. The uncaging light pulse was adjusted in duration (from 0.2 to 0.4 ms) and intensity (from 10 to 20 mW under the objective) to produce a response similar to a unitary EPSP in the soma (0.2–0.8 mV). These values cover the range of somatic recordings of physiological unitary EPSPs under optically confirmed activation of one individual spine on a neuron (Magee and Cook, 2000; Nevian et al., 2007; Bloodgood et al., 2009; Enoki et al., 2009).

At the end of each experiment, a detailed morphological reconstruction of dye-loaded neurons was carried out on a stationary upright microscope (AxioExaminer D1 with zoom tube (0.5–4x), Carl Zeiss Microscopy LLC) equipped with a high spatial resolution CCD camera (1392 × 1024 pixels; Pixelfly-qe, PCO Imaging, Kelheim, Germany) mounted on a spinning-disc confocal scanner (Yokogawa CSU-10). At the end of every experiment, this system collected z-stacks of confocal images for the detailed morphological reconstruction of basal dendrites and spines. The morphological reconstruction was used to confirm that (a) the recorded spine was spatially isolated so that no other spine was closer than 5 μm from the uncaging light spot in both x,y and z-dimensions, and (b) to determine the distance of the recording site from the soma. In addition, the recorded length of the basal dendrite was used to calibrate the optical signal in terms of membrane potential using the known bAP amplitude at the corresponding distances (see below).

### Data analysis

Membrane potential optical signals related to uEPSP followed by bAPs were recorded typically for 60 ms at a frame rate of 5 kHz at a near physiological temperature of 32–34°C. Data were analyzed and displayed using the NeuroPlex program (RedShirtImaging), written in IDL (Exelis Visual Information Solutions, Boulder, CO) and custom Visual Basic routines. Background fluorescence can be a significant determinant of ΔF/F signal size. Raw data were first corrected for this effect by subtracting the average background fluorescence intensity determined from an unstained area on the slice. Subsequently, signal alignment software was used to correct temporal jitter in AP initiation and possible small preparation movements during averaging (Popovic et al., 2015a). In the temporal domain, AP signals were aligned by cross-correlation of the electrically recorded APs in each trial to the reference signal acquired at the start of averaging. In the spatial domain, camera images were aligned offline in two dimensions by image cross-correlation to compensate for possible small lateral movements of the preparation (Popovic et al., 2015b). The correct focus of the image in the z-dimension was verified before each trial; minor adjustments were often necessary. The spatially and temporally aligned signals were averaged, and slow changes in light intensity due to the bleaching of the dye were corrected by dividing the data by an appropriate dual exponential function derived from the recording trials with no stimulation (Grinvald et al., 1982). The residual slow changes in baseline after bleaching correction, if present, had no effect on uEPSP and bAP amplitude and waveform because they were small and approximately 100 times slower than the rising phase of an action potential (Supplementary Figure S3; Foust et al., 2010; Holthoff et al., 2010; Popovic et al., 2011, 2015a). The waveform of the AP signal was reconstructed from a set of data points using Cubic Spline Interpolation, a piecewise continuous curve passing through each data point (Popovic et al., 2011). Subthreshold optical signals were calibrated on an absolute scale (in mV) by normalizing to an optical signal from a bAP, which has a known declining amplitude along basal dendrites, as previously determined by patch-pipette recordings from basal dendrites (Nevian et al., 2007; Palmer and Stuart, 2009). The reported uncertainties in bAP amplitudes do not influence the spine/dendrite EPSPs ratio. This method of calibration produces the same results as normalizing signals to optical recordings corresponding to long hyperpolarizing pulses delivered to the soma, which attenuate relatively little as they propagate along dendrites (Stuart and Spruston, 1998; Palmer and Stuart, 2009; Holthoff et al., 2010).

## Results

### Majority of spine synapses are not electrically isolated from basal dendrites

The first series of experiments confirmed our previous findings in L5 cortical neurons (Popovic et al., 2015a) and determined that prior conclusions are valid for other principal cortical pyramidal cell classes. The experiments were conducted on basal dendrites of L2/3, L5, and L6 pyramidal neurons. The only direct way to determine the degree of electrical isolation of spine synapses is to simultaneously record EPSP signals from the spine head and the parent dendrite at the base of the spine following selective quantal activation of a single synapse. Under these conditions, the evoked EPSP signal (EPSP_*spine*_) in the spine head represents a voltage drop caused by the synaptic current flow (I_*syn*_) across the constant Ohmic electrical resistance of the spine neck (R_*neck*_) and the electrical input impedance of the dendrite (Z_*dendrite*_) connected in series according to the expression:


(1) EPSP=spineI(R+neckZ)dendritesyn

The EPSP signal in the parent dendrite at the base of the spine represents a voltage drop caused by the same synaptic current flow across the Z_*dendrite*_ alone, according to the expression:


(2) EPSP=dendriteIZsyn(KochandZador,1993)dendrite.

**FIGURE 1:**
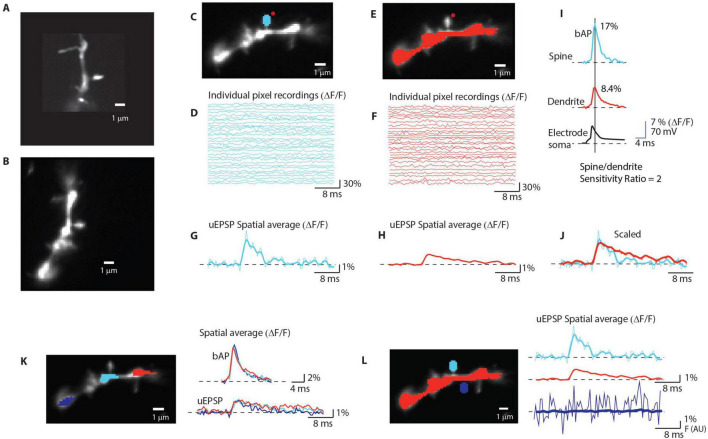
Optical recording from individual dendritic spines. **(A)** High magnification image of a spine on a basal dendrite; confocal z-stack projection. **(B)** A high-magnification single-frame image of a spine in recording position was obtained with the CCD for voltage imaging. **(C)** Selection of pixels receiving light from the spine head (blue). Red dot: position of the uncaging light spot. **(D)** Recordings of fractional fluorescence light intensity changes (ΔF/F) from 31 individual pixels (labeled in blue in **D**) during an evoked uEPSP. Temporal average of 8 trials; uEPSP optical signals cannot be resolved in single-pixel recordings. **(E,F)** The same explanation for **(C–E)** applies to recordings from a subset of individual red pixels receiving light from the dendrite region at the base of the spine. **(G,H)** Spatial averages of signals from blue pixels **(G)** and red pixels **(H)** reveal the size and shape of uEPS P in spine head and in parent dendrite respectively. Thin line: row data. Thick line: data filtered by one pass of the 1-2-1 binomial smoothing routine. **(I)** Spatial average of bAP signals from blue (spine head) and red (dendrite) pixels used to determine the sensitivity ratio and normalize signals from spine head and parent dendrite. **(J)** The scaled uEPSP signals from the spine head and dendrite are similar. **(K)** Spatial average of bAP and uEPSP signals from 3 regions along the parent dendrite. **(L)** Recordings from spine head (blue), dendrite (red), and a region without a spine (blue) at an identical distance from the dendrite as the spine head. The effect of light scattering from the dendrite is not detectable.

According to these two expressions, the experimental approach to the electrical role of spines is conceptually simple, even though it is technically demanding to the degree that prevented these measurements for several decades. If one can simultaneously record EPSP_*spine*_ and EPSP_*dendrite*_ at the required spatiotemporal resolution, one can anticipate two different categories of results. One possible outcome is that the EPSP amplitude is significantly larger in the spine head than in the parent dendrite. This result would imply a relatively large R_*neck*_ comparable to Z_*dendrite*_ at a given dendritic location. For example, if the amplitude of the recorded EPSP_*spine*_ is two times larger than EPSP_*dendrite*_, the result would indicate that R_*neck*_ is equal to Z_*dendrite*_. The other possible result is that EPSP_*spine*_ and EPSP_*dendrite*_ are similar in size and shape. A direct from this type of result would be that R_*neck*_ must be negligible compared to Z_*dendrite*_.We used two-photon uncaging of glutamate and organic intracellular voltage-sensitive dye recordings to monitor quantal uEPSP signals simultaneously from individual dendritic spine heads and parent dendrites at the base of the spine in three classes of principal cortical neurons, L2/3, L5, and L6 pyramidal cells in somatosensory brain slices. The results documented that, at near-physiological temperature, the method has adequate sensitivity at the required spatiotemporal resolution to record and faithfully reconstruct individual uEPSPs and APs signals separately from spine heads and parent dendrites. Figure 1 illustrates a typical experiment. A high magnification confocal fluorescence image (confocal z-stack projection) of a dendritic spine on a basal dendrite of an L5 pyramidal neuron labeled with a voltage-sensitive dye JPW3028 (Materials and methods) is shown in Figure 1A. Figure 1B shows a single frame image focused on the same spine in the recording position obtained with the CCD for voltage imaging. Figures 1C,D illustrate optical recordings of the evoked subthreshold (uEPSP) signals from the spine head adjusted in uncaging light intensity and duration (Materials and methods) to mimic quantal glutamate release resulting from the arrival of one AP at the presynaptic bouton. Individual pixels that receive light from the spine head are labeled in blue (Figure 1C). Optical recordings from individual blue pixels indicate that the available sensitivity in terms of the signal-to-noise ratio (S/N) is insufficient to resolve uEPSP signals (Figure 1D). Figures 1E,F illustrates the simultaneous recordings of the same evoked uEPSP signal from a random subset of individual red pixels, which receive light from the section of the parent dendrite at the base of the spine. Again, the recordings from individual pixels that receive light from the parent dendrite cannot resolve optical signals related to the small uEPSP voltage transient. Figures 1G,H illustrate the powerful effect of spatial averaging. The spatial average of signals from both blue and red pixels covering the spine head and the parent dendrite, respectfully, increases the number of collected photons, improves the S/N, and reveals the size and shape of the uEPSP signal. Following the uEPSP, we evoked a bAP by a brief depolarizing current pulse delivered from the somatic patch electrode.

The bAP-related signal was recorded optically as spatial average of the same set of blue and red pixels receiving light from the spine head and the parent dendrite (Figure 1J). In all measurements, we used the bAP-related optical signals to normalize the sensitivity of optical recordings from different locations (scaled signals). This normalization is based on prior knowledge that bAP has the same size and shape in the spine and the parent dendrite. Thus, it can be used as a calibration standard to calibrate optical signals in terms of membrane potential (Palmer and Stuart, 2009; Holthoff et al., 2010; Popovic et al., 2014). Scaling of optical signals from different locations, which, as a rule, have different surface-to-volume ratios and, hence, different recording sensitivities, is required to compare signals correctly. Figure 1J illustrates that, in the typical experiment, the scaled uEPSP signals from the spine and the parent dendrite are not different after allowing for the noise in the recording.

**FIGURE 2:**
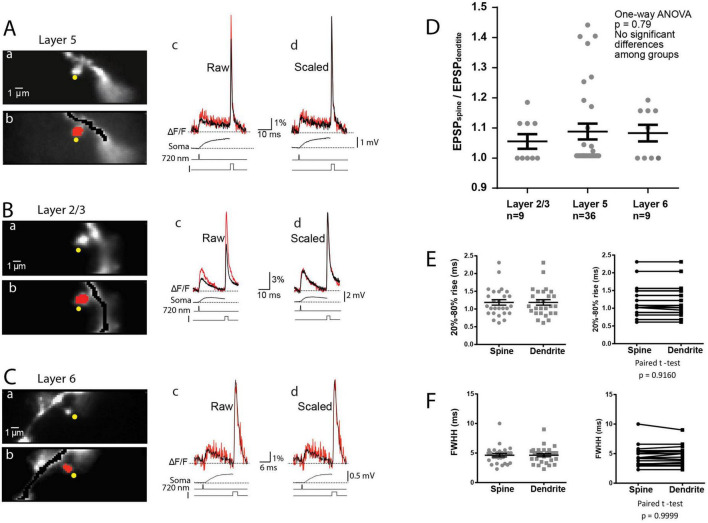
Optical recordings of EPSP and AP signals from individual spines and parent dendrites from cortical L5, L2/3, and L6 pyramidal neurons. ***A*,** L5 pyramidal neuron. **(A)** The fluorescence image of a spine in the recording position was obtained with the CCD for voltage imaging. The yellow dots indicate the position of the 720 nm light spot ∼0.6 μm in diameter, used for two-photon glutamate uncaging. **(B)** Selection of pixels used for the spatial average of optical signals from the spine head (red) and parent dendrite (black). **(C)** Evoked uEPSP and AP signal from the spine head and parent dendrite superimposed. Bottom three black traces: Top: electrode recording of somatic uEPSP. Middle: the uncaging command pulse. Bottom: transmembrane current pulse delivered by a somatic patch electrode. **(D)** Superimposed signals from the spine head and parent dendrite were corrected for recording sensitivity differences by normalizing recordings to the bAP optical signal. **(B,C)** Similar recordings from L2/3 and L6 neurons, respectively. **(D)** Scatter plot of individual values of the ratio (uEPSPspine/uEPSPdendrite) for the three classes of cortical pyramidal neurons. Vertical lines show mean ± SEM. **(E,F)** Scatter plot of individual values of uEPSP 20–80% rise time **(E)** and of uEPSP full width at half height (FWHH) **(F)**. Vertical lines show mean ± SEM.

The described improvements in the sensitivity of optical recordings by spatial averaging over short distances are justified because, according to multicompartmental numerical simulations, the electrical length constant of the dendrite will make the surface membrane area of both the ∼1 μm spine head and the ∼10 μm long section of the dendrite at the base of the spine very nearly isopotential for all plausible biophysical parameters (Popovic et al., 2014, 2015a). This was confirmed experimentally as illustrated in Figure 1K. In Figure 1K, the amplitudes of bAP and uEPSP signals from 3 regions along the parent dendrite are compared and found to be the same, indicating that the section of the parent dendrite covered by red pixels in Figure 1E is very nearly equipotential. We further confirmed (Figure 1L) that there is no significant crosstalk between signals from the spine head and the dendrite in the superficial layers of the slice. The measurements show the absence of any signal from a location without a spine (dark blue pixels) at the same distance from the dendrite as the spine head (light blue pixels). This result demonstrates that scattered light from the dendrite did not contribute to the uEPSP signal from the spine. The summary data in Figure 2 show that, in the preponderance of cases, there was very little or no detectable difference in both the amplitude and the kinetics between EPSP_*spine*_ and EPSP_*dendrite*_. We did not detect any cases where the dendritic uEPSP signal was significantly smaller than the spine signal with all amplitude differences due to the random shot noise in optical recordings. In all such cases, the ratio EPSP_*spine*_/EPSP_*dendrite*_ was assumed to be 1. This result implies that R_*neck*_ is negligible compared to Z_*dendrite*_. The same result was obtained from L2/3, L5, and L6 pyramidal neuron measurements. Combined with our previous data from L5 pyramidal neurons (Popovic et al., 2015a), the summary plot in Figure 2D shows that negligible R_*neck*_ was confirmed in 9 spines from L2/3 neurons, 36 spines from L5 neurons, and 9 spines from L6 neurons. The average EPSP_*spine*_/EPSP_*dendrite*_ ratio from all experiments was 1.08 ± 0.03 (*n* = 54), with no significant difference between the three classes of neurons (One-way ANOVA; *p* = 0.79). The distribution of individual values for the kinetics of EPSP_*spine*_ and EPSP_*dend*_ (rise time and FWHH) are shown in Figures 2E,F. While the average ratio of 54 experiments was close to 1.0, we recorded several outliers with a ratio as high as 1.4. This result is in line with a finding that a small percentage (< 5%) of spines is characterized by high and spontaneously reversible diffusional resistance (Bloodgood and Sabatini, 2005) (see Discussion). Our results from basal dendrites of three different classes of principal cortical neurons provide direct evidence that, in most mushroom spines, R_*neck*_ is negligible relative to Z_*dendrite*_. We conclude that synapses on these spines are not electrically isolated from the parent dendrite to the degree that would imply functional meaning. The interpretation of reported results combined with control experiments did not require specific assumptions. The standard methodological controls in voltage imaging, including linearity in fractional fluorescence light intensity changes with membrane potential, the absence of pharmacological effects of the JPW3028 dye, the lack of photodynamic damage under correct experimental conditions, as well as the absence of significant effects of the slow bleaching of the dye minimized by correction procedures, have been analyzed in detail and repeatedly documented in both pioneering studies (Ross et al., 1977; Grinvald et al., 1982) and our earlier reports (Djurisic et al., 2004; Canepari et al., 2007; Foust et al., 2010; Holthoff et al., 2010; Popovic et al., 2011, 2015a). Considering these control experiments, it is unlikely that pharmacological effects or photodynamic damage caused by the dye specifically and exclusively affected R_*neck*_. However, it was not possible to rule out this possibility. It is also unlikely that the spines of the selected neurons in the upper layer of the slice were somehow altered and not representative. Again, we could not rule out this possibility. At the same time, as described above, the estimates derived from measurements of diffusional resistance of the spine neck carried out on pyramidal neurons in brain slices agree with our conclusions. In addition to the established methodological controls described above, we carried out experiments to address two possible sources of errors that could influence the accuracy of our measurements and the validity of our conclusions. These include (1) the contribution of extrasynaptic receptors and (2) inadequate spatial resolution of voltage imaging.

### Contribution of extrasynaptic receptors

**FIGURE 3:**
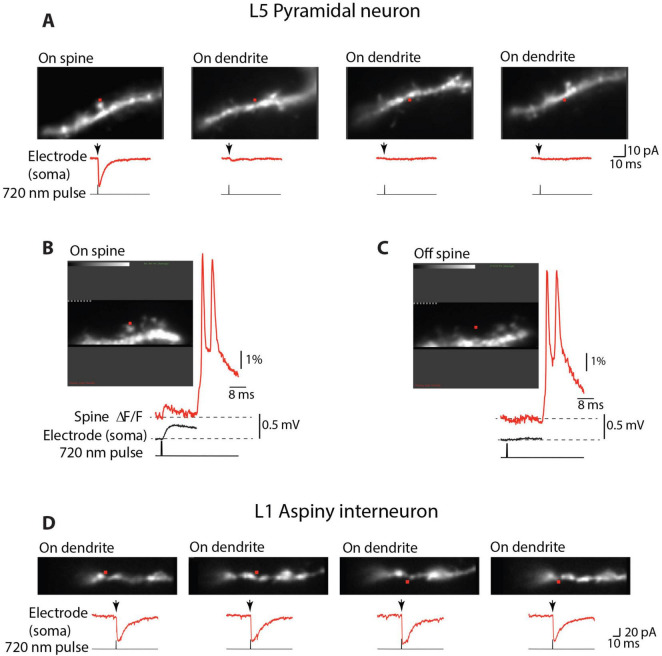
Selective activation of individual synapses**. (A)** Uncaging glutamate on smooth, aspiny regions of basal dendrites of L5 pyramidal neurons (On dendrite) evoked currents (red traces recorded under voltage-clamp) that were either not detectable or represented a tiny fraction of the current evoked by activating synapses on neighboring spines (On spine). Red dots indicate the position of the 720 nm light spot used for two-photon glutamate uncaging. **(B)** Uncaging glutamate on an individual spine evokes clear uEPSP as revealed by voltage imaging (red trace) and electrical recording from the soma (black trace). **(C)** Uncaging glutamate at a distance from aspiny dendritic regions equivalent to the distance of the spine head failed to produce measurable uEPSP responses in both optical and electrical recordings. **(D)** Uncaging glutamate on aspiny dendrites on L1 interneurons evoked a fast and clear response at all tested locations.

The validity of our results depends on the spatial selectivity of the two-photon uncaging of glutamate. If the two-photon glutamate release on spine head synapses also significantly activated extrasynaptic receptors on parent dendrites, this effect would contribute to the recorded similarity of responses from spines and dendrites. Both pioneering and the most recent studies firmly established single spine spatial resolution of two-photon glutamate uncaging (Matsuzaki et al., 2001; Smith et al., 2003; Tazerart et al., 2020). It has been documented that the non-specific activation of glutamate receptors on parent dendrites is so small that it can be safely neglected (Matsuzaki et al., 2001; Sobczyk et al., 2005; Popovic et al., 2015a). We confirmed this conclusion in voltage-clamp experiments (Figure 3A), showing that uncaging glutamate on aspiny membrane regions of basal dendrites of pyramidal neurons at the range of light intensities used in our measurements (Materials and methods) evoked currents that were either not detectable or represented a tiny fraction (4 ± 0.9%; *n* = 34) of the current evoked by activating synapses on neighboring spines. Moreover, voltage imaging under the current clamp revealed that uncaging glutamate at a distance from the aspiny dendritic regions of pyramidal neurons equal to the distance of the spine head failed to produce any measurable uEPSP responses (Figures 3B,C). This result was consistently confirmed in 9 experiments. In contrast to these results on pyramidal neurons, and as a positive control, we found that uncaging glutamate directly onto the membrane of aspiny dendrites on L1 interneurons evoked a fast and clear response at all locations tested in *n* = 18 interneurons (Figure 3D). This result is in line with previous reports showing that AMPA glutamate receptors are mainly absent from extrasynaptic regions on basal dendrites of cortical pyramidal neurons (Carter and Sabatini, 2004; Sobczyk et al., 2005; Higley and Sabatini, 2012; Popovic et al., 2015a) while they are widely distributed along densely innervated smooth dendrites of aspiny interneurons (Gulyás et al., 1999; Sancho and Bloodgood, 2018).

### Light scattering and spatial resolution

Light scattering in the brain tissue limits the spatial resolution of voltage imaging in wide-field microscopy mode. The crosstalk caused by light scattering from the parent dendrite to the spine head was shown previously to be small in superficial layers of the slice (< 10%; see Figure 3 in Popovic et al., 2014, 2015b) and without a significant effect on our conclusions. The lack of significant contribution of scattered fluorescence light from the dendrite was confirmed again in the present experiments (Figure 1L). Furthermore, the crosstalk in the opposite direction, from spine heads to the parent dendrites, was even smaller and often not detectable, primarily due to the significant difference in size, as documented before (< 3%; see Figure 3 in Popovic et al., 2014, 2015b).

It is probably helpful to reiterate that the ratio (uEPSPspine/uEPSPdendrite) does not depend on absolute values of R_*neck*_ and Z_*dendrite*_ and, therefore, does not require calibration of optical signals in terms of membrane potential. Accordingly, possible errors due to unavoidable inaccuracies in calibrating optical signals in terms of membrane potential (Popovic et al., 2015a; Acker et al., 2016; Kwon et al., 2017; Cornejo et al., 2022) can safely be ruled out. Taken together, the data argue that uEPSPs are not significantly attenuated as they propagate from synapses on spine heads to the parent dendrite and imply that, in electrical terms, synapses on explored cortical dendritic spines behave in the same way as synapses made directly on dendrites.

### Temporal summation at single spines

**FIGURE 4:**
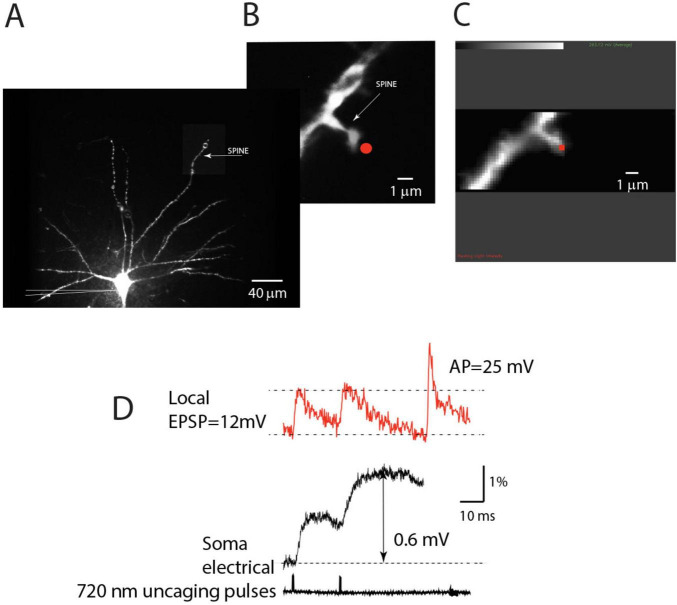
Contribution of individual synapses: experimental design. **(A)** An individual neuron is labeled with a voltage-sensitive dye. An isolated spine is identified in a distal region of basal dendrite under low magnification. A patch electrode (shown schematically) is attached to the soma for electrical recording and stimulation. **(B)** Fluorescent image of an isolated spine in recording position obtained at high magnification with a high-resolution CCD. **(C)** The image of the same spine obtained by reading out a 24 × 80 pixel subsection of the CCD camera for voltage imaging at 5 kHz. The red dot indicates the position of the 720 nm light spot used for two-photon glutamate uncaging. **(D)** Red traces: optical recordings of local uEPSP signals evoked by two-photon glutamate uncaging, followed by a bAP signal evoked by a depolarizing pulse delivered by the somatic patch electrode. Optical signals are spatial averages of all bright pixels in the image. Black traces: somatic electrode recordings (upper) and uncaging command pulses (lower).

The ability to monitor local electrical signaling from individual spines and parent dendrites allowed us to record and analyze the temporal summation of uEPSPs following repetitive activation of single spine synapses. Because presynaptic neurons often fire in a burst (Kole, 2011), these experiments mimic physiological conditions in which the natural sensory stimulus activates isolated individual spines on dendritic branches *in vivo* (Jia et al., 2010; Chen et al., 2011; Varga et al., 2011). Figure 4 illustrates the experimental approach for monitoring temporal summation at individual synapses. A fluorescence image of an L5 pyramidal neuron labeled with the voltage-sensitive dye was used to identify an isolated spine close to the surface of the slice (Figure 4A). Using patterned illumination based on the CGH system (Materials and methods), a 720 nm light spot from a pulsed laser was positioned within 0.5 μm from the edge of an individual spine for two-photon uncaging of glutamate. A patch electrode was attached to the cell body to monitor the membrane potential and synaptic currents. The electrode also allowed us to pass a depolarizing current and evoke a bAP used to calibrate optical signals on an absolute scale (Materials and methods).

**FIGURE 5:**
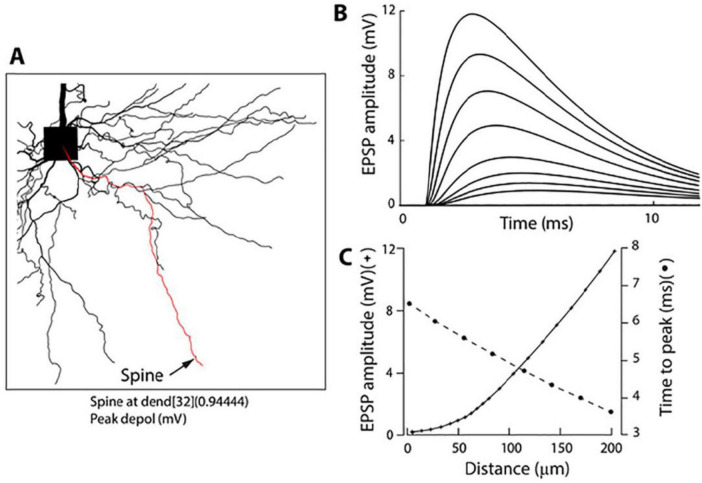
Numerical simulation (NEURON 6.0) of the electrotonic spread of an EPSP along a basal dendrite. **(A)** Morphometrically detailed reconstruction of basal dendrites obtained with a high-resolution spinning disk confocal microscope. The location of a spine at the distal end of a basal dendrite is indicated by an arrow. **(B)** Shape and size of an EPSP from multiple locations at increasing distances from the site of origin (spine) along the basal dendrite as indicated in **(C). (C)** EPSP amplitude and time-to-peak as a function of distance from the soma.

The uncaging light pulse was adjusted in duration and intensity to mimic a unitary EPSP in the soma (0.2–0.8 mV; Materials and methods). To optimize sensitivity in this set of experiments, uEPSP-related optical signals were recorded at the site of origin as the spatial average of the spine and a small region (∼12 μm) of the dendrite at the base of the spine. We showed above (Figures 1, 2) that this entire surface is very nearly isopotential. Optical signals were recorded simultaneously with the electrode recording from the soma (Figure 4D). From this type of measurement, we established that an average quantal uEPSP recorded at the site of origin had a 20–80% rise time of 1.2 ± 0.07 ms and FWHH of 4.6 ± 0.3 ms (*n* = 26). Scatter plots showing the distribution of individual values are shown in Figures 2E,F. Due to the rapid kinetics of EPSPs at the site of origin, there was very little or no temporal summation of signals at the synapse if the uncaging pulses were delivered with an inter-pulse interval ≥ 20 ms (Figure 4D). However, a clear summation was recorded at the soma-axon region. This is because EPSP in the soma had slower kinetics and considerably reduced amplitude (Figure 4D). This result is expected due to the well-known electrotonic propagation and RC filtering effect on subthreshold electrical signals in the dendritic cables (Rall et al., 1967). We confirmed the declining electrotonic propagation and the RC filtering effect on the dynamics of EPSPs in basal dendrites using numerical simulation (Figure 5). Morphometrically detailed reconstruction of basal dendrites has been obtained with a high-resolution spinning disk confocal microscope. The location of a spine at the distal end of a basal dendrite is indicated by an arrow (Figure 5A). Figures 5B,C illustrates the gradual change in shape and size of an EPSP computed at multiple locations at increasing distances from the site of origin (spine) along the basal dendrite. A detailed computational model is made available^1^ (Djurisic et al., 2008; Popovic et al., 2014, 2015a).

**FIGURE 6:**
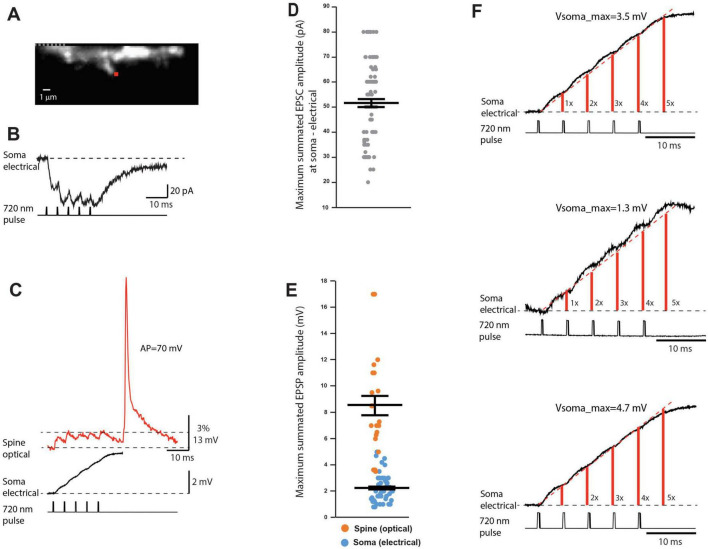
Summation of uEPSP signals. **(A)** Fluorescence image of a spine in recording position. A red dot indicates the position of the 720 nm light spot used for two-photon glutamate uncaging. **(B)** Upper trace: summed uEPSCs recorded with a somatic patch electrode under voltage-clamp in response to repetitive glutamate uncaging at 200 Hz. Lower trace: command uncaging pulses. **(C)** Upper trace: Summating uEPSP-related signal recorded optically from the dendritic spine and the parent dendrite section shown in **(A)** in response to repetitive glutamate uncaging at 200 Hz. Middle trace: Summating uEPSP signal recorded with a somatic patch electrode simultaneously with the optical recording of local signals. Bottom trace: Uncaging command pulses. **(D)** Scatter plot of individual voltage-clamp measurements of maximum summed uEPSC amplitudes in response to 5 uncaging pulses at 200 Hz. **(E)** Scatter plot of maximum summed uEPSP amplitudes in response to 5 uncaging pulses at 200 Hz as recorded optically from the spine and electrically from the soma. **(F)** Magnified display of uEPSP trains recorded with a somatic patch electrode (as in middle trace in **C**), indicating three typical examples of consistent linear summation of subthreshold signals at the soma-axon region.

To ensure that local summation will occur, the following measurements were carried out with 5 uncaging pulses delivered at 200 Hz, mimicking the burst of APs in a presynaptic neuron. At the start of the experiment, we made somatic electrical recordings of uEPSCs under voltage clamp in response to repetitive activation of a spine synapse. Figure 6A shows an image of a spine in recording position with a red dot indicating the location of the 720 nm light spot used for two-photon glutamate uncaging. Figure 6B illustrates a typical response showing that synaptic currents caused by individual uncaging pulses summed in a pronounced sublinear fashion.

A plateau was reached after the second pulse in this experiment, and the maximum current during the plateau phase reached a peak value of 70 pA. The summary data from an extensive series of similar measurements show that the average maximum current amplitude during the plateau phase was 52 ± 1.7 pA (*n* = 94). The distribution of individual values is shown in the scatter plot (Figure 6D).

The voltage-clamp measurement of synaptic current was followed by optical recording of local uEPSPs in current-clamp mode, evoked by an identical uncaging protocol from the same spine on the basal dendrite. Figure 6C indicates a typical example of a prominent sublinear summation of local uEPSPs consistent with the summation profile of synaptic currents. The maximum response was reached after the second EPSP in this experiment, with the local depolarization reaching a plateau at 6 mV. At the same time, the summation of attenuated uEPSP signals measured at the soma with a patch electrode was nearly linear, resulting in the maximum somatic depolarization of 3.5 mV. In a series of measurements of this kind, the average peak amplitude of the summed uEPSP at the site of origin was 8.8 ± 1.0 mV (*n* = 23). The corresponding summed uEPSP peak amplitude in the soma was 2.3 ± 0.2 mV (*n* = 56). The distribution of individual values of the summed uEPSP in the spine and the soma is shown in the scatter plot in Figure 6E. The RC filtering effect of basal dendrites and the cell body promotes an approximately linear summation at the soma-axon region by slowing the kinetics of EPSP signals as they propagate to the soma. Figure 6F shows three additional typical examples of linear summation of the train of uEPSPs as recorded in the soma with a patch electrode. An important advantage of this biophysical property of neurons is that it provides a mechanism for a wide dynamic range of near-linear integration of synaptic signals at the soma-axon region (the output end of the neuron) while minimizing saturation of the driving force for synaptic current at remote local synapses on spines (the input end of the neuron).

**FIGURE 7:**
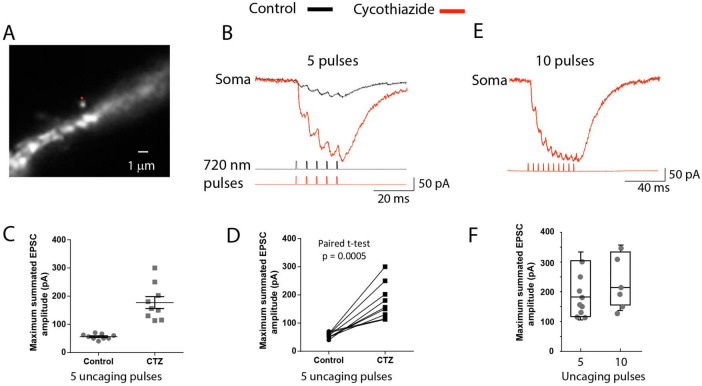
AMPAR desensitization underlies the sublinear summation of unitary uEPSCs. **(A)** Fluorescence image of a spine in recording position. The red dot indicates the position of the 720 nm light spot used for glutamate uncaging. **(B)** Summing uEPSCs recorded with a somatic patch electrode under voltage-clamp in response to repetitive glutamate uncaging at 200 Hz under control conditions (black traces) and following bath application of 100 μM of AMPAR desensitization inhibitor cyclothiazide (red trace). Cyclothiazide caused a dramatic increase in the maximum synaptic current response. Bottom traces: Uncaging command pulses**. (C)** Scatter plot showing the distribution of data from individual experiments. **(D)** Paired *t*-test. **(E)** Summating uEPSCs recorded with a somatic patch electrode under voltage-clamp in response to 10 glutamate uncaging pulses at 200 Hz following bath application of cyclothiazide. **(F)** Comparison of mean plateau values of the summed EPSC following 5 and 10 uncaging pulses.

Because individual synapses are critical computational units in the nervous system, it is important to investigate biophysical properties determining the sublinear temporal summation of subthreshold signals in dendritic spines. It is clear that relatively small local dendritic depolarization caused by summed uEPSP train (< 10 mV; Figure 6E) cannot explain the pronounced sublinear temporal summation based on the reduction of a large driving force for sodium ions (V_Na_ = 60.60 mV), the dominant current carriers underlying EPSPs (Miyazaki and Ross, 2022). Additionally, glutamate receptors on the postsynaptic side are far from saturation during quantal transmission (Liu et al., 1999; McAllister and Stevens, 2000). Thus, it is likely that AMPA receptor desensitization (Kiskin et al., 1986) plays an important role in the sublinear summation of unitary uEPSPs shown in Figure 6. All glutamate receptors undergo desensitization. This process is fast in AMPA receptors, occurring with a time constant of several milliseconds, and can generate greater than 90% decrease in current amplitudes within 20 ms. The effect does not depend on EPSP amplitude (Traynelis et al., 2010). To test the prediction that AMPA receptor desensitization plays an important role in the sublinear summation, we monitored uEPSCs summation under voltage-clamp as recorded by a patch-electrode at the soma under control conditions and after AMPA receptor desensitization was inhibited by 100 μM cyclothiazide (CTZ) (Partin et al., 1993). Figure 7A illustrates a fluorescence image of a spine on a basal dendrite of L5 pyramidal neuron in recording position. CTZ is known to produce inhibition of glutamate receptor desensitization (Fucile et al., 2006). On average, adding 100 μM cyclothiazide (Figure 7B) increased the maximum synaptic current response from 52 ± 2.1 pA to 177 ± 21 pA (*n* = 9), an increase in the mean value of 340% (Figure 7C).

**FIGURE 8:**
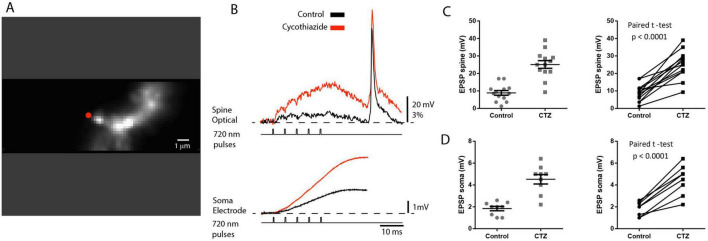
The effect of desensitization on the summed train of uEPSPs. **(A)** Fluorescence image of a spine in recording position. The red dot indicates the position of the 720 nm light spot used for two-photon glutamate uncaging. **(B)** Upper traces: optical recordings of local summed uEPSP signals from the spine and parent dendrite under voltage-clamp evoked by repetitive glutamate uncaging at 200 Hz. Black trace: control conditions. Red trace: bath application of 100 μM AMPAR desensitization inhibitor cyclothiazide. Lower traces: simultaneous somatic patch electrode recordings. Bottom traces: uncaging command pulses. **(C)** Left panel: scatter plot of individual values of maximum summed uEPSP measured optically in the spine under control conditions and following bath application of cyclothiazide. Right panel: paired *t*-test shows a significant difference. **(D)** Left panel: scatter plot of individual values of maximum summed uEPSP measured with somatic patch electrode under control conditions and following bath application of cyclothiazide. Vertical lines show mean ± SEM. Right panel: paired *t*-test indicates a significant difference.

The scatter plot of the summary data in Figure 7C shows the distribution of individual values. The effect was clear and statistically significant, as indicated by the paired *t*-test in Figure 7D. In CTZ, with desensitization abolished, the synaptic current responses still summate in a sublinear mode. However, the maximum current amplitude level is shifted toward larger values. In these conditions, the plateau is likely caused by receptor saturation. One would expect all synaptic AMPA receptors to be activated at saturation, so a further application of glutamate will not significantly increase the synaptic current. Indeed, this result was obtained when the train was extended from 5 to 10 uncaging pulses (Figure 7E). The mean plateau value of the summed EPSC following 10 uncaging evoked releases of glutamate reached a value of 218 ± 36 pA, a slight increase that was not statistically significant (*t*-test, *p* = 0.48, *n* = 6) (Figure 7F). Following the somatic EPSC recordings in the voltage-clamp mode, we determined the effects of the desensitization block on the train of local EPSPs measured optically as the spatial average of the activated dendritic spine and a small section of the parent dendrite at the base of the spine (Figure 8A). Because the recordings were carried out from the same location, normalizing the sensitivity was not needed, and the results were directly compared. The local EPSP response measured optically in the spine and a small dendritic section at the bottom of the spine increased from 8.4 ± 0.3 mV in the control solution to 25 ± 3.4 mV (*n* = 13), an increase of the mean value of 297%, following bath application of cyclothiazide (Figure 8B). On average, the cyclothiazide caused a 244% increase in summed EPSP response in the soma, from 1.8 ± 0.2 mV to 4.4 ± 0.4 mV (*n* = 9). The scatter plot of the summary data (Figures 8C,D) shows the distribution of individual data and the high statistical significance of the effect.

## Discussion

### EPSP transfer from the spine to the dendrite

The optical recording data show that the electrical resistance of the spine neck in most of the examined L5 pyramidal neurons under physiological conditions is too low, by a factor of 10 or more, relative to the input impedance of the parent dendrite, to cause a voltage drop that would contribute significantly to the amplitude of the EPSP in the spine head. Similar experiments on L2/3 and L6 pyramidal neurons indicated that the same conclusion is valid for the two additional classes of principal cortical neurons. These results rule out the electrical role of the examined mushroom spines on basal dendrites of cortical pyramidal neurons. Our findings are based on measurements of the amplitude ratio (AR) of optical signals AR = EPSP_spine_/EPSP_dend_ = 1 + (R_neck_/Z_dend_) rather than on an attempt to measure absolute values of R_neck_ and Z_dendrite_. The recorded values of the functional parameter AR, which controls the synaptic weight, are, on average, very close to 1, indicating that R_neck_ is negligible relative to Z_dendrite_. Our measurements also revealed the existence of a small subset of spines with a ratio as high as 1.4 (Figure 1D). Strong evidence for a small percentage of spines (∼5%) characterized by transitory and spontaneously reversible (on a minutes’ scale) high diffusional isolation of spine heads corresponding to high R_neck_ had been reported earlier (Bloodgood and Sabatini, 2005). Our study could not examine the stability of high AR over time in these rare cases because the repetition of temporal averaging of signals is limited by photodynamic damage. The specificity of these spines is presently not apparent. At present, there is no evidence that the electrical input impedance of dendrites may vary to an extent that would significantly modify the recorded AR and, hence, the electrical role of spines.

Our results agree with early theoretical predictions (Rall, 1974; Koch and Zador, 1993) and our numerical simulations with an entire range of plausible biophysical parameters (Popovic et al., 2014, 2015a) as well as with the initial voltage-sensitive dye study of subthreshold signals from dendritic spines carried out using low-sensitivity confocal imaging in combination with numerical simulations (Palmer and Stuart, 2009). The data also agree well with the early (Svoboda et al., 1996) and more recent (Bloodgood and Sabatini, 2005; Takasaki and Sabatini, 2014; Tønnesen et al., 2014) measurements, which showed low diffusional resistance of the spine neck corresponding to low R_neck_. Particularly solid experimental evidence based on diffusional resistance, which supports low R_neck_ is the measurement of Na^+^ ion flux, which mediates the rapid removal of sodium from the spine head (Miyazaki and Ross, 2017, 2022). Due to the close analogy between diffusional coupling and the electrical resistance, it is difficult to argue against the strong implications of Na^+^ diffusional measurements for the upper bound of the possible electrical resistance of the spine neck. Our results imply that mushroom spines on thin basal dendrites are characterized by uniform electrical behavior regardless of considerable natural morphological variations (Jones and Powell, 1969; Tønnesen et al., 2014). Thus, the data argue that relatively small changes in the morphology of individual spines known to occur following induced synaptic plasticity (Takasaki et al., 2013; Araya et al., 2014; Tønnesen et al., 2014; Tazerart et al., 2020) are likely to be the by-product and not the cause of plastic changes.

The results supporting low R_neck_ call into question several existing hypotheses regarding the electrical role of spines. One group of studies based on indirect Ca^2+^ measurements postulated that spine neck filters EPSPs (Araya et al., 2006b,^2014^) and that spines amplify EPSPs up to 45-fold, thus facilitating electrical interactions among coactive inputs and promoting associated forms of plasticity and storage (Harnett et al., 2012). Other studies, however, based on the same Ca^2+^-measurements technique, contradicted these interpretations and conclusions. A report on the electrical behavior of spines in mitral cells of the olfactory bulb is an example of a striking lack of correlation between spine neck lengths and the uncaging-evoked EPSP amplitude, rise time, and associated Ca^2+^ signals in the spine head (Bywalez et al., 2015). A similar study based on Ca^2+^ imaging combined with morphological STED data and diffusional FRAP measurements from spines of CA1 pyramidal neurons reported a similar lack of correlation between spine dimensions (neck length and diameter) on the one hand and evoked EPSP amplitudes in the soma and Ca^2+^ transient in the spine head on the other hand (Takasaki and Sabatini, 2014). The reported lack of correlation is consistent with R_neck_ varying with neck dimensions within a range of values much smaller than Zdendrite. The inconsistencies in indirect evidence could be due to Ca^2+^ signals being slow and highly non-linear indicators of transmembrane voltage changes. Additionally, depolarizing Ca^2+^ currents through NMDARs and other voltage-dependent channels provide positive feedback, and the non-linear relationship between calcium signals and transmembrane voltage is unstable due to the high sensitivity of the state of calcium channels to the history of the resting membrane potential. These factors make it difficult to accurately extrapolate from repeatedly evoked Ca^2+^-imaging signals to membrane potential transients without electrical measurements.

Another group of studies combined voltage imaging data with numerical simulations and deconvolution procedures to determine the electrical role of spines (Acker et al., 2016; Kwon et al., 2017; Cartailler et al., 2018; Lagache et al., 2019). They interpreted their results as supporting the hypotheses that R_neck_ plays a vital role in determining the spine EPSP amplitude and that spine geometry plays a key role in shaping the EPSP time course. In an *in vivo* study using two-photon microscopy and a genetically encoded protein voltage probe, Cornejo et al. (2022) concluded that spines are isolated voltage compartments that could be important for dendritic integration and disease states. However, none of the studies in this group had adequate sensitivity and spatiotemporal resolution to detect and reconstruct EPSP signals simultaneously from the spine head and the parent dendrite. Additionally, as a rule, the results of numerical simulations and deconvolution procedures in neurophysiology depend on assumptions and approximations. Therefore, the interpretation of these results is uncertain. Our optical recordings of electrical signals at two ends of the spine do not support the above hypotheses.

### Contribution of an individual synapse to electrical signaling

Using high-sensitivity voltage imaging, we obtained unique data on the temporal summation of unitary EPSPs at the site of origin, single excitatory synapses on dendritic spines. Our study shows that (1) Temporal summation of repetitive quantal EPSP inputs at the site of origin (spine head) is markedly sublinear; (2) AMPAR desensitization seems to be one of the important determinants of sublinear EPSP summation. Due to the complexity of cyclothiazide effects (e.g., Fucile et al., 2006), the exact role of desensitization in sublinear EPSP summation would require a separate study. The reduction of the synaptic driving force likely plays a minor role because local depolarization at the synaptic site is small compared to the equilibrium potential for Na ions. (3) Distinctly sub-linear uEPSP summation at the synaptic site is paralleled by near-linear summation at the soma-axon region; (4) Repetitive activation of individual synapses on examined spines did not initiate APs at the soma-axon region or a dendritic NMDA spike, independently of the input frequency.

In conclusion, local individual uEPSPs have fast kinetics, resulting in little or no temporal summation at frequencies below 100 Hz. At a higher frequency (200 Hz), unitary EPSPs summate locally in a pronounced sub-linear manner. At the soma-axon region, EPSPs at both low and high frequencies summate in a near-linear fashion due to the broadening of the EPSP signal caused by RC filtering in the dendrite. Thus, our study shows that the sublinear summation of EPSP at the synaptic sites prevents depolarization buildup and synaptic saturation. This mechanism widens the dynamic range of near-linear summation of repetitive EPSPs at the soma-axon region.

An earlier review article (Zecevic, 2023) is an overview of the results from several laboratories, summarizing some of the arguments described here.

## Data Availability

The raw data supporting the conclusions of this article will be made available by the authors, without undue reservation.

## Footnotes

1
https://modeldb.science/2016666

## Supplementary material

**Supplementary Figure S1** Patterned holographic illumination was recorded from a thin layer of rhodamine 6G dispersed on a microscope slide. The 720 nm spot covered ∼3 pixels, corresponding to a size of ∼600 nm.

**Supplementary Figure S2** Stimulated emission depletion (STED) effect of the uncaging 720 nm red light reducing the intensity of voltage-sensitive dye fluorescence excited by 532 nm excitation light. Upper traces: raw signals from the dendrite and spine, and superimposed scaled signals. Bottom black trace: uncaging command pulse. Lower traces: superimposed scaled signals on an expanded time scale. Bottom black trace: uncaging command pulse. The short (0.2 ms) uncaging pulse controlled by a Pockels cell modulated only one data point during the 5 kHz recording of the voltage-sensitive dye signals. The peak amplitudes of the uEPSP and bAP were not influenced by the STED effect.

**Supplementary Figure S3** The absence of residual dye bleaching effect on uEPSP and bAP signals shape and size. **(A)** Optical recordings of uEPSP and bAP signals from the spine head and parent dendrite. Duration of uEPSP and bAP upstroke indicated by arrows. **(B)** Baseline optical signals from the start of recording to the start of the uEPSP on an expanded time scale. Note that, during a period equivalent to the upstroke of the uEPSP and bAP, changes in signal amplitude due to the residual bleaching after bleaching correction (Materials and methods) are smaller than the noise in recordings. We conclude that residual bleaching did not affect signal amplitudes.
